# Supplementary material for: Are carbon emissions trading and green financial instruments synergistic? -Comprehensive quantitative research based on content analysis
Source: PLoS One. 2024 Mar 7;19(3):e0298601. doi: 10.1371/journal.pone.0298601 (PMC10919601; doi:10.1371/journal.pone.0298601)
Supplement: S1 Data — (ZIP) [file pone.0298601.s001.zip › Data/地级市Z_F工作报告.zip!/╔╜╢1⁄2/╝├─■╩╨/└┤╫╘╣1⁄2╓┌║┼í╛┬φ┐╦╩2╛▌═°í┐_2015.docx]

各位代表：

现在，我代表济宁市人民政府向大会报告工作，请予审议，并请市政协委员和其他列席会议的同志提出意见。

一、2014年工作回顾

过去的一年，面对复杂多变的经济形势，在中共济宁市委的坚强领导下，全市上下深入学习贯彻党的十八大和十八届三中、四中全会精神，以习近平总书记视察山东视察济宁重要讲话精神为指引，坚持稳中求进、改革创新，全市经济社会发展取得新成绩。地区生产总值完成3800亿元、增长9.6%；公共财政预算收入334.2亿元、增长10.6%；固定资产投资2538.2亿元、增长16%；社会消费品零售总额1664.1亿元、增长12.8%；城市居民人均可支配收入30428元，农民人均纯收入12650元，分别增长8.8%和11.5%，居民消费价格指数控制在3.2%，较好地完成了年度任务目标。

（一）结构调整取得积极进展。工业经济提质增效，完成工业投资1357.3亿元、增长22.8%、高于固定资产投资增幅6.8个百分点，新兴产业和高新技术产业投资增幅分别达到27.6%和34.5%。规模以上工业增加值增长11.7%、高于全省平均2.1个百分点，制造业增加值占比达到59.3%、拉动工业增长7.4个百分点。高新技术产业产值占比提高1.5个百分点。新增规模以上企业470家，4家企业上榜中国企业500强。现代农业扎实推进，实施粮食高产创建工程，粮食总产达到115.8亿斤，实现“十一连增”。新扩建农产品质量安全示范园区93个，新增市级农业产业化龙头企业97家、家庭农场431家，土地流转面积达到190.5万亩，农业机械化水平提高到87%。渔业转型升级和畜牧业生态健康养殖成效明显。全国小农水重点县建设实现全覆盖。全国春季农业生产现场会在我市召开，粮食高产创建整建制推进和大田作物土地托管服务模式在全国推广。服务业扩量提速，完成增加值1444.2亿元、占比提高1.1个百分点，实现税收增长13.5%、占比达到40.5%、同比提高3.1个百分点。新增主辅业务分离列统企业1320家、居全省首位。实现旅游社会总收入442亿元、增长12.6%。金融服务能力不断增强，年末存贷款余额分别达到3691.6亿元、2563亿元，同比增加130.5亿元、286.5亿元。8家村镇银行开业运营，小额贷款公司发展到22家。新增新三板等挂牌企业6家，企业债券融资142.8亿元、同比增加33.5亿元。快速高效处置企业担保圈风险，维护了区域金融安全。县域经济争先进位，倍增计划深入实施，公共财政预算收入增长11.8%，高于全市平均1.2个百分点，金乡县、梁山县公共财政预算收入实现倍增，泗水县、邹城市列入省县域经济科学发展试点县。

（二）重点领域改革实现突破。大力推进简政放权，建立行政权力清单和收费清单制度，分两批取消下放市级行政审批事项111项，免征市级行政事业性收费105项。市县政府机构改革基本完成。深化农村土地制度改革，87.8%的村完成土地确权登记颁证，供销社改革有序展开。扎实推进财税金融体制改革，市直部门预算和“三公经费”预决算全部向社会公开，县级“三公经费”预决算公开试点扩大至9个县市区。农村信用社改革全面启动，国家级小微企业信用体系试验区获批设立，普惠金融覆盖面持续扩大。积极推进工商注册制度改革，新登记市场主体8.36万户、居全省首位。

（三）创新驱动战略深入实施。全市研发经费支出占GDP的比重达到1.55%。济宁高新区荣获“中国产学研合作创新示范基地”称号。如意集团获批国家纺纱工程技术研究中心，成为我市第一家国家级研发平台。新增院士工作站7家、省级以上企业技术中心14家、国家级科技孵化器2家、孵化器面积40万平方米。中科院计算所济宁分所、省科学院济宁分院等研发机构加快建设。强化人才引领作用，引进国内外高层次人才2984人，国家千人计划专家达到16人、泰山学者特聘专家达到24人。科技创新能力显著增强，取得重要科技成果206项，专利授权4012件，实现技术交易额8.15亿元；石墨烯材料、生物酶制剂、光纤传感等科技专项取得重大突破，实现产业化生产。

（四）城乡面貌发生显著变化。城镇化率提高1.93个百分点、达到50.26%。中心城区功能日趋完善，改造棚户区434.9万平方米，竣工保障性住房4908套。完成38条背街小巷整治任务，新增天然气用户1.37万户、供热面积200万平方米。新建过街天桥10座，1万辆公共自行车成为一道靓丽风景。城市展示馆、济宁园博园建成开放。济宁客运西站投入使用，万达广场等城市综合体开业运营。泗河综合治理开发启动实施，“三河六岸”改造提升等牵动性工程加快推进。“五城同创”活动深入开展，顺利通过省级文明城市验收。都市区一体化加速推进，编制完成《都市区空间战略与行动计划》，组织管理、产业布局、道路交通、生态建设等全方位融合，区划调整效应逐步显现。新开通至鱼台、金乡、汶上城际公交。济徐高速济宁至鱼台段、滨河大道济宁至梁山段、运河航道北延等重点工程加快实施。美丽乡村建设全面启动，县城和小城镇公共服务、基础设施水平不断提升，17个示范片区建设亮点纷呈，农村环境综合整治任务提前两年完成，城乡环卫一体化、市场化实现全覆盖。生态环境建设成效明显，实施蓝天、碧水、增绿工程，建成大气污染防治项目175个，控制建筑工地和工矿区周边扬尘、取缔城区小型燃煤炉具、淘汰黄标车等取得突破，大气环境质量综合改善幅度居全省第3位。主要河流水质稳定达标，南水北调续建配套工程年度任务如期完成。保护修复湿地26.6万亩，治理塌陷地2万亩，新增造林26.8万亩、国家级生态乡镇29个。

（五）文化强市首善之区建设快速推进。大力弘扬优秀传统文化，广泛开展社会主义核心价值观教育活动，村居“四德榜”实现全覆盖，我市被授予全省“四德工程”建设示范市，一批优秀文艺作品在全省、全国获奖。大幅提升公共文化服务水平，建成127个乡镇综合文化站、5893个村文化大院、3492个村文化广场。开展“千场大戏进农村”、“万场演出惠民生”和农村公益电影放映等活动共计10万余场。市文化中心启动建设。大运河济宁段进入世界文化遗产名录。深入实施文化突破曲阜战略，曲阜文化经济特区核心区《发展战略策划》编制完成，孔子学院总部体验基地批准设立，孔子博物馆主体工程竣工。文化产业加快发展，经营单位超过1万家，从业人员10万人，在建文化产业项目98个，尼山圣境、水浒文化园等一批文化产业项目进展顺利。

（六）民生保障水平全面提升。全市民生支出260亿元，占财政支出的56%。为民所办10件实事如期完成。实施“万人创业助推工程”，新增城镇就业6.5万人、高校毕业生就业2.7万人、农村劳动力转移就业7.9万人。社会保障体系不断完善，在全省率先实现五项社会保险制度全覆盖，连续第十年调整提高企业退休人员养老金标准，城乡低保标准分别提高45元、57元，农村互助养老数量居全省首位，适度普惠型儿童福利保障制度纳入全国试点。“先看病、后付费”诊疗服务模式不断完善，“万名保健医生进农户”覆盖所有行政村。计划生育利益导向政策全面落实。中小学标准化建设步伐加快，5个县市区通过“县域义务教育发展基本均衡县”国家评估验收，全市农村教学点基本达到省普通小学办学标准。农村专项扶贫扎实推进，8.1万贫困农民实现脱贫；较好解决了21万农村居民饮水安全问题。安全生产持续平稳，食品药品监管不断强化，应急管理切实加强，治安防控体系日益完善，社会保持和谐稳定。第十一届村民委员会换届选举工作顺利完成。11个对口援疆项目全部建成。圆满完成全国第三次经济普查工作任务。国防动员、民兵预备役和双拥工作富有成效。国家安全、新闻出版、广播影视、民族宗教、公共资源交易、住房公积金、外事侨务、耕地保护、统计、物价、粮食工作取得新成绩。档案史志、人防地震、水文气象、妇女儿童、老龄、慈善、红十字等项事业加快发展。

一年来，我们以开展党的群众路线教育实践活动为抓手，全面加强政府自身建设。严格落实中央“八项规定”和省、市委实施办法，聚焦反对“四风”，整改问题40项，化解信访积案263件，新建修订公务接待等制度26项。加强法治政府建设，主动接受人大依法监督、政协民主监督，人大代表建议和政协提案办复率均为100%。严格落实党风廉政建设责任制，自觉遵守各项廉政纪律，深入开展“双评”和纠风专项治理，加强审计监督，严肃查办了一批违规违纪案件，树立了风清气正、务实高效的政府形象。

各位代表，过去的一年，在经济下行压力加大、矛盾困难增多、发展任务艰巨的严峻形势下，我们同心协力，奋发作为，办成了一些大事、好事，在一些事关济宁长远发展的重大问题上取得积极进展。一是对上争取实现重大进展。国务院领导对我市资源型城市转型工作作出重要批示，曲阜中华优秀传统文化传承创新示范区、国家级生态保护与建设示范区等重大战略，有望进入国家层面。省政府制定出台支持济宁可持续发展16条意见。一批国家和省重大基础设施项目相继在我市布局，“国家采煤塌陷地复垦治理研究中心”设立建设，鲁南城际客运专线纳入国家规划，跨湖高速、机场迁建等项目进入省级层面推进实施。二是招商引资实现新突破。继惠普项目落户之后，甲骨文人才产业基地、中兴智慧城市产业园、中科智造信息产业园、软通动力信息产业生态圈等一批高端信息产业项目集群式落户我市，150家IT企业跟踪入驻，信息产业呈现裂变式发展态势。新引进世界500强企业3家、达到46家，实际利用外资8.9亿美元，对外投资12.1亿美元、居全省首位。对接全国工商联执委会、央企等系列招商活动成效显著。三是成功举办第23届省运会等系列重大活动。精心做好市场开发、赛会运行、环境整治、舆论宣传等各项工作，圆满完成各项赛事活动，兑现了省运会、残运会“同城举办、同样精彩”的庄严承诺。高水平承办国际孔子文化节、世界文明论坛、世界儒学大会，进一步强化了济宁国际儒学研究交流中心地位。

各位代表，过去一年各项成绩的取得来之不易，是省委、省政府和市委正确领导的结果，是市人大、市政协和社会各界监督支持的结果，是全市上下共同努力的结果。在此，我代表市人民政府，向全市各族人民群众、各界人士、驻济部队官兵和公安干警致以崇高敬意！向各级人大代表、政协委员，各民主党派、工商联、各人民团体，向支持参与济宁建设发展的中央、省驻济单位和港澳台同胞、海外侨胞、国际友人表示诚挚感谢！

我们也清醒地认识到，经济社会发展中还存在不少困难和问题。经济下行压力依然较大，县域经济发展不平衡，产业结构仍然不合理；中小微企业融资难、融资贵问题突出，区域性金融风险依然存在；生态环境建设任务艰巨，空气环境质量与群众期盼还有较大差距；重点领域的改革任务繁重，体制机制还存在不适应的问题；有些政府工作人员法治意识淡薄，作风建设有待加强，个别部门单位还存在办事效率低、不作为、慢作为、乱作为的问题。对此，我们一定高度重视，采取切实措施加以解决。

二、2015年重点工作

今年是全面深化改革的关键之年，是全面推进依法治国的开局之年，也是全面完成“十二五”规划的收官之年。我国经济发展已经进入新常态，发展速度、发展方式、发展动力都发生重大变化，这对我们推进发展提出了新的更高要求。我市改革发展进入攻坚期，既充满严峻挑战，也面临诸多有利条件。中央和省对济宁的发展高度重视，出台了支持我市转型发展的有关政策，布局了许多事关济宁长远发展的大项目、好项目；我市一系列重大战略部署全面实施、纵深推进，诸多历史性机遇正加速转化为现实发展优势，一批高端高质项目建成投产，优势主导产业集群加速形成，支撑发展的基础更加坚实；改革红利充分释放，全市广大干部群众干事创业的热情高昂，成为推动发展的新动能。只要我们准确把握新常态，积极适应新常态，以新思维、新作为全力做好政府各项工作，就一定能够创出新业绩、开拓新局面！

今年工作的总体思路是：深入贯彻党的十八大和十八届三中、四中全会精神，以习近平总书记系列重要讲话、视察山东视察济宁重要讲话和重要批示精神为指引，积极主动适应经济发展新常态，坚持稳中求进工作总基调，坚持工业化、城镇化“两化并进”，抓住用好“六大机遇”，突出改革开放、转方式调结构、创新驱动、社会治理“四个加大”，更大力度突破工业、城市、县域、文化、生态“五大领域”，完善提升民生保障“十大体系”，努力开拓经济社会持续健康发展新途径，为实现打造鲁西科学发展高地、建设淮海经济区中心城市、与全省同步提前全面达小康“三大目标”奠定坚实基础。主要预期目标为：地区生产总值增长9%左右；公共财政预算收入增长10%左右；规模以上工业增加值增长10.5%左右；固定资产投资增长15.5%左右；社会消费品零售总额增长13%左右；外贸进出口增长6%左右，实际利用外资稳步增长；城镇居民人均可支配收入增长9%左右，农民人均纯收入增长10%左右；城镇登记失业率控制在4%以内；居民消费价格指数涨幅控制在3%以内；全面完成省下达的节能减排约束性目标；人口自然增长率控制在9.5‰以内；城镇化率提高2个百分点。

完成上述目标任务，必须深刻把握经济发展新常态的内涵和规律，打破思维惯性，坚定不移推动工作指导重大转变；必须加大产业结构调整力度，推动传统产业向中高端迈进，增强现代制造业和新兴服务业支撑作用，提高经济发展质量效益；必须始终把改革作为强大动力，尊重和发挥地方、基层、群众首创精神，大胆探索，勇于实践，依靠改革增活力添动力；必须牢固树立法治理念，运用法治思维和法治方式推动发展、化解矛盾、维护稳定，全面推进依法治市；必须提高科技创新能力，大力实施创新驱动战略，优化区域创新环境，力促经济发展动力从传统增长点转向新的增长点；必须注重保障改善民生，着力推动更多公共资源、公共服务向基层延伸，让全市人民共享改革发展成果。

今年重点抓好九个方面的工作。

（一）加快工业化信息化深度融合，打造工业经济升级版。坚持把工业经济作为转型升级主战场，加快结构调整、产品升级，提升工业经济核心竞争力，加速新型工业化进程。

举全市之力培育信息产业。抓住我市信息产业膨胀裂变的重大机遇，提速推进惠普、中兴通讯、甲骨文、大唐高鸿、中科智造、软通动力等高端信息产业项目建设。密切与国内外IT产业巨头的合作，加快形成产业孵化、人才实训、软件开发测试等新型业态，率先在软件和信息服务业、电子信息制造业上实现新突破。支持济宁高新区、任城区等信息产业园区建设，促进产业项目、高端人才、研发机构、创投基金加速集聚，努力打造全省IT产业新“硅谷”。

把提升传统产业作为工业强市主战场。综合运用科技创新、技术改造、信息融合、市场拓展、兼并重组等手段，推动传统产业提质增效。加快制造业向智能工厂、智能生产转型步伐，打造大数据、云计算等公共平台，拓展3D打印、数字制造等技术应用，培育发展个性化、智能化、网络化的新业态。对工程机械、纺织服装、汽车及零部件、化工、食品、医药等10大优势产业，逐个确定年度目标和提升计划，鼓励企业“腾笼换鸟”、“空间换地”、“电商换市”，统筹推进化解过剩产能工作。重点支持100个重大技改项目，完成技改投资800亿元，新增规模以上工业企业200家以上。

以打造产业集群促进县域经济提档升级。抓好主导产业集群规划，明确产业发展方向，引导产业集群式布局。强化设计、技术、人才、信息、投融资等服务功能，为产业集群发展提供增值服务。突出抓好装备制造、专用车制造、农用机械、食品加工、纺织服装、新能源等产业集群的整合提升，以打造完整产业链为目标，有针对性地引进和培育上下游关联企业，形成由龙头企业引领、中小企业配套跟进的优势产业集群。

着力优化民营经济发展环境。抓好专业特色镇村、创业园区建设，建成16个中小企业创业园。加快市县两级中小企业公共服务中心建设，在技术创新、项目孵化、人才引进等方面，为中小企业提供全方位服务。发挥产业引导基金、投资基金、直接债务融资作用，切实解决中小微企业融资难、融资贵问题，确保中小微企业贷款增速不低于各项贷款平均水平、总量不低于上年同期水平。实施“百千万培训工程”，办好“民营经济大讲堂”，打造有知识、会管理、善经营的新型企业家队伍。全面落实扶持民营经济政策，持续掀起全民创业热潮，全力推动民营经济大发展，让济宁成为最富生机的创业热土、最具活力的发展高地！

（二）加快转变农业发展方式，大力发展现代农业。以土地经营权确权登记颁证为契机，创新农业生产组织方式，推进农业生产经营集约化、标准化、专业化，努力实现农业增效、农民增收。推进农业生产方式现代化。深化农业产学研合作，加快济宁国家农业科技园规划建设，依托农业龙头企业开展技术攻关，引进和推广新品种、新技术、新成果。提升农业机械化水平，主要农作物力争实现全程机械化。合理引导先进生产要素进入农业产业化领域，支持“云农场”等电子商务平台建设，打造农业发展新的增长极。发展多种形式适度规模经营。扩大农村产权流转交易市场建设试点，规范土地经营权有序流转，推动农业适度规模经营。大力培育新型经营主体，研究制定农民合作社发展规范、家庭农场基本标准和工商资本进入农业领域监管办法。完善农业社会化服务体系，推广大田作物托管服务模式，鼓励各类社会服务组织发展壮大。年内新增市级农业产业化龙头企业50家、家庭农场500家、农民合作社1000家。提升农产品质量水平。推进农业标准化生产，发展品牌农业、特色农业和高端优质农业。加强农业综合执法，健全农产品质量安全监管体系，整建制开展县乡农产品质量安全监管示范创建，新扩建农产品质量安全示范区90个，新增标准化生产基地80万亩、“三品一标”认证70个。夯实农业可持续发展基础。实施粮食高产创建示范工程，确保粮食总产稳定在100亿斤以上。健全农技推广体系，培育新型职业农民。加快水利基础设施建设，完成7694项农田水利和小水源工程，建设高标准农田32万亩，建成4万亩智能化节水灌溉示范区，治理水土流失60平方公里。全面推进精准扶贫工作，确保让每一名困难群众的生活更有盼头、更有尊严！

（三）加快发展现代服务业，提升对经济发展的支撑作用。坚持把服务业作为转方式调结构的战略重点，有效增加服务业投资，提升服务业发展质量，力争投资增长20%，增加值、税收占比提高2个百分点。

激发生活性服务业发展活力。做好文化与旅游融合发展文章，突破体制机制障碍，整合文化旅游资源，推动有条件的景区景点率先实行统一管理、集中开发、整体运营。深化与港中旅等大型旅游集团的战略合作，提升水浒文化园和太白湖、峄山、羊山、宝相宫、南阳古镇等景区景点层次水平，加快尼山圣境、兴隆文化园等重点项目建设，支持微山湖创建5A级景区，丰富修学游、乡村游等旅游产品，放大儒家文化、运河文化旅游品牌效应，努力打造国内外知名旅游目的地城市。顺应互联网经济发展新趋势，规划建设多功能、多业态的电子商务园区，吸引一批国内外大型电子商务企业区域总部、营运中心落户济宁，培育一批行业电子商务平台，实现线上线下联动发展。鼓励发展健康养老、医疗保健、体育休闲、会展节庆、教育培训和社区家政等服务业，培育新的消费增长点。

提升生产性服务业助推功能。现代物流业，抓好铁水联运、内河集装箱运输和临港物流交易服务中心规划建设，加快兖州北站物流园、嘉祥和梁山铁水联运物流园、钢联电商产业园等项目建设进度，打造以京杭运河为依托、物流园区为支撑、电子商务为平台的鲁西南物流集散中心。金融保险业，积极引进银行、保险、证券等金融机构入驻，支持地方金融机构加快发展，推动保险业参与社会管理、服务保障民生。完善金融市场体系，鼓励银行机构创新信贷产品，优化信贷结构，密切银企合作，有效扩大信贷和表外融资规模，鼓励有条件的企业上市或发行债券直接融资，提升金融对实体经济发展的支撑作用。科技服务业，以研发设计、技术转移、创业孵化、知识产权为重点，促进科技与经济深度融合。抓住知名软件企业落户我市机遇，推动企业与高校联合开展人才培训，努力打造区域人力资源中心，优化人力资本结构，提升人才服务经济发展的能力。

强化服务业发展政策支持。降低市场准入门槛，鼓励社会资本参与交通、通信等基础设施建设，进入市政公用事业领域，参与文化、教育、卫生、体育等社会事业建设。严格落实服务业用电、用气、用水、用热与工业同价政策，提高服务业建设用地比例。积极实施政府向社会购买公共服务，逐步扩大购买服务范围。支持服务业平台建设、人才培养引进、品牌培育提升，吸引社会资本、生产要素加速向服务业集聚，力促服务业发展提速、结构优化，努力把服务业打造成我市最具潜力和活力的支柱产业！

（四）加大创新型试点城市建设力度，增强经济增长的内生动力。提高企业创新发展能力。启动企业创新“331”工程，支持和引导企业加大研发投入、建立研发机构，密切与科研院所战略合作，积极承担国家、省重大科技专项，加快突破核心关键技术，增强企业核心竞争力。集中打造10个创新集群、新增10家以上创新示范企业、100家科技型小巨人企业、3000家科技型小微企业，高新技术产业产值增长15%。提升平台研发服务水平。加快国家纺纱工程技术研究中心、山东科学院济宁分院、工业机器人智能应用技术研究院、华大基因济宁分院等公共创新服务平台建设，依托中科院计算所济宁分所、深圳先进技术研究院济宁分院、鲁南工程技术研究院等行业平台，聚集一批高端人才，孵化一批科技创新成果，形成支持重点产业转型升级的新动力。完善科技金融服务体系。以天使基金培育初创期企业，以风险投资支持成长期企业，以股权直接投资成熟期企业，以银行信贷全链条支持产业发展，推进企业创新链、产业链和资金链加速融合。打造科技金融服务平台，多渠道引进金融机构和各类基金，以政府资金引导和撬动社会资金，有效解决科技企业融资难题。抓好高层次人才培养引进。大力实施海外人才引进“511”计划和国内人才集聚“百千万”工程，启动实施“鲁西科学发展高地人才支持计划”，吸引更多国家千人计划专家、泰山学者等高层次领军人才和团队来我市创新创业。坚持招商引资与招才引智并重，以项目聚集人才，用人才支撑项目，实现人才、项目、产业的互促共进。完善引才、用才、留才政策，落实企业股权、期权等激励机制。深化高新区人才管理改革试验区建设，发挥“人才联盟”作用，搭建好人才公共服务平台，畅通人才服务绿色通道，为打造鲁西科学发展高地提供坚实智力支持！

（五）全面深化改革扩大开放，持续释放发展生机活力。大力推进改革攻坚。加快转变政府职能，进一步简政放权，做好清权、减权、优权、制权各个环节的工作，着力清理部门“红头文件”，最大限度向市场、社会和基层放权。深化行政审批制度改革，建立健全行政审批事项目录清单，能放的全部下放到县市区，该取消的全部取消。加快行政类事业单位分类改革，完成事业性质中介机构转企改制，引导社会组织承接政府转移的部分公共服务职能。全面完成市县公务用车制度改革。深化县级公立医院综合改革，范围扩大至所有县市区。基本完成农村土地承包经营权确权登记颁证工作，稳步推进农村集体经济组织产权制度改革试点。深入推进财政体制改革，加快改革预算管理制度，建立透明的全口径预算体系，年内市县政府全面公开政府预决算，所有使用财政资金的部门均公开部门预决算和“三公”经费预决算。优化财政支出结构，清理规范财政专项资金，盘活财政存量资金，集中财力保障重点领域特别是民生支出。加大对县市区一般性转移支付力度，逐步减少县市区地方配套，提升基层政府保障能力。转变财政支持方式，设立10亿元产业发展引导基金，更好地发挥财政资金的杠杆作用。严格控制地方政府各类债务增长，逐步把地方政府债务纳入全口径预算管理。加快金融改革发展，全面推进普惠金融和小微企业信用体系试验区建设，稳妥发展小额贷款公司、民间融资机构，扩大企业直接融资规模。深化农村信用社改革，推动设立县域农商行、社区银行、村镇银行、金融超市。加快济宁资本中心建设，争取设立齐鲁股权交易鲁南运营中心。强化金融监管，有效防范和妥善化解区域性金融风险，维护良好金融生态环境。稳步开展国有企业股权多元化改革，健全公司法人治理结构，规范国有企业负责人薪酬管理，加快建立以管资本为主的国有资产监管模式，鼓励发展混合所有制经济，支持社会资本参与国有企业改制重组。

提升对外开放水平。积极融入国家“一带一路”、自贸区建设等发展战略，构筑更具活力的开放型经济体系。坚持高水平“引进来”，进一步转变招商引资方式，注重理念契合、文化融合，发挥企业主体作用，着力加强与世界500强企业、大型央企、知名民企的战略合作，以信息产业、先进制造业、现代服务业为主攻方向，突出抓好以商招商、园区招商、产业链招商，力争在招商引资上取得新突破。大力开拓国际市场，引导企业开展国际商标注册、国际质量体系认证，加快品牌建设，打开对外投资和贸易通道。精心组织“千企百展”活动，扩大机电设备、食品医药、纺织服装等主导产业产品出口规模，巩固拓展日本、韩国、东盟和非洲、南美等国家和地区市场。积极争取设立济宁综合保税区，推进金乡直通场站、青岛保税港区邹城功能区规划建设。鼓励兖州煤业、如意集团等龙头企业加大境外市场开发力度，努力打造一批源自济宁的跨国公司。

（六）提升城镇化内涵品质，统筹推进城乡一体发展。坚持以“人”的城镇化为核心，完善城市总体规划和都市区专项规划，加快构建城乡互促、共同繁荣的发展格局，建设区域中心城市。

以板块融合加速都市区发展。坚持规划引导、基础先行、交通一体，大力实施都市区空间战略与行动计划，优化城市空间布局，明晰功能定位，统筹安排产业园区、公共基础设施项目，形成区域融合发展格局。加大任城区城中村开发建设和棚户区、老旧小区改造力度，完善供热、供气等功能，着力突破现代服务业和新兴产业，发挥好中心城区辐射带动作用。提升太白湖新区教育、医疗、文化等公共服务功能，强化主导产业培育，抓好高科技产业园、国家地理信息产业园、生态文化教育基地等规划建设，推进新区建设与产业发展互促并进，倾力打造生态新城和城区主中心。坚持以产业西进带动城市西跨，引导优势资源、优质项目、优秀人才向西部加速流动，推进商贸物流、装备制造等重点产业项目和科技孵化中心、天河云计算中心等功能性项目建设，提升产业综合实力和产城融合水平，打造西部经济增长极。支持济宁高新区创新体制机制，整合人力资源、公共技术、科技金融等服务平台，建设区域性产学研中心，提升特色主导产业，壮大战略新兴产业，培育高科技服务业，构建创新驱动发展生态体系，打造创新型科技新城。加速东部大型工业聚集区建设，以兖州工业园区和邹城开发区为主阵地，引导产业互补发展，增强区域协作配套能力，推动资金、技术、人力资源跨区域密集交流。以路网互通互联、公共交通一体布局为牵动，加快城际基础设施建设，开工北二环东延工程。加快济徐高速济宁至鱼台段建设，完成滨河大道济宁至梁山段主体工程，滨湖大道南延开工建设，做好济宁机场迁建、跨湖高速前期工作，推进鲁南客运专线规划建设。完成京杭运河航道北延长沟、邓楼船闸建设，实现京杭运河黄河以南全线通航。有序推进泗河综合治理开发，实施引汶补源入济工程。加快兖州区在道路、医疗、教育、广电、公交等方面与中心城区同城一体、深度融合。

以精细化管理提升城市形象。深化城市管理体制改革，整合市政、园林、环卫、执法管理资源，推动管理重心下移，构建多方联动、齐抓共管城市管理新格局。以“五城同创”活动为抓手，重拳治理“脏乱堵”，新建和改造一批环卫和垃圾处理设施，治理背街小巷40条。推行公共服务市场化改革，鼓励社会力量通过委托授权、特许经营参与城市建设管理。加快“智慧城市时空云平台”建设，建成集地理信息、交通管理、市容环境等于一体的智能化信息系统，提升城市管理现代化水平。

以服务功能提升推进镇村建设。进一步优化资源配置，促进公共财政向镇村倾斜，公共设施向镇村布局，逐步完善镇村服务功能，提升公共服务均等化水平。加快镇驻地和农村道路、供排水、污水处理、供暖供气等公共设施建设，提高镇村污水处理率、供热率和燃气普及率。纵深推进美丽乡村建设，实施增绿、增亮工程，加快山区、道路、村庄绿化，建设绿色庭院、生态廊道，打造绿树成荫的自然景观，确保村庄主要街道、公共活动场地照明实现全覆盖。强化生态文明村连线成片整治，实施一村翠绿、一塘清水、一处广场、一院洁净工程，确保全市25%的村建成生态文明村。加快美丽乡村示范片区建设，开展改厕改灶等14项重点工作，以实实在在的行动，托起自然、文明的美丽乡村梦！

（七）加快生态济宁建设，提升生态文明水平。以建设国家生态保护与建设示范区为统领，以绿化、治水、治气系列工程为抓手，加快国家环保模范城市创建步伐，力争建成全国绿化模范城市。

打好大气污染防治持久战。实施生态攻坚“十大行动”，严格执行逐步加严的区域大气污染物排放标准，试点火电行业废气超低排放，巩固扩大脱硫脱硝除尘成果，铁腕治理燃煤电厂不达标排放。深化小型锅炉专项治理，推广燃煤清洁生产技术，严格控制挥发性有机物污染，有效解决工业异味问题。推行大气污染防治网格化管理，扩大“高污染燃料禁燃区”范围，重拳整治城市和工矿区扬尘、机动车尾气、露天烧烤、秸秆焚烧等污染顽症。严格落实烟花爆竹限放措施。完成6.39万辆黄标车淘汰任务。纵深推进“十大重点绿化工程”，新增造林20万亩、绿色通道300公里，全市森林覆盖率达到30%，让群众呼吸到更清新的空气、享受更多的蓝天白云！

打赢水污染防治攻坚战。扎实做好南水北调输水通道工程维护、水质安全、环境保护等各项工作，保证一泓清水永续北送。完善“治、用、保”防控体系，完成107个重点治污项目，严厉打击污水直排、重金属污染等环境违法行为，提高考核断面水质质量，实现老运河、洸府河、泗河等重点河流稳定达标。推进环南四湖大生态带建设，大区域展开采煤塌陷地治理布局，保护修复湿地10万亩，确保淮河治污迎查再创佳绩！

打胜节能减排阵地战。以保障区域生态安全为目标，严格执行污染物排放许可和总量控制制度，以生态环境容量倒逼产业结构调整。制定工业项目负面清单，加强固定资产投资项目节能评估和审查，严控“两高”行业扩张和产能过剩行业新上项目。大力发展节能环保产业，实施100个重点节能降耗项目，培育循环经济发展新模式，综合利用先进节能技术、合同能源管理等有效措施，大力压减万元GDP能耗、主要污染物排放量，确保完成“十二五”节能减排任务！

（八）全力突破文化建设，打响“文化济宁”城市品牌。济宁是中华文明的重要发祥地和儒家文化的发源地，在中华文化大格局中占有特殊地位，理应担负起弘扬中华优秀传统文化的重大责任和历史使命。加快首善之区建设。全面启动优秀传统文化六进普及、道德提升、文化惠民等“七大工程”，构建市县图书馆“尼山书院”、乡村儒学讲堂、国学阅览室等儒家文化传播网络，深入开展全民阅读活动。创新孔子文化节、尼山论坛举办形式，丰富活动内容，办好第七届世界儒学大会，提高济宁世界儒学交流中心地位。深入推进全国文明城市创建，大力弘扬社会主义核心价值观，提升群众文明素养，激发全社会向上向善的良好道德风尚。推进曲阜文化经济特区建设。按照战略策划方案，加快建立特区决策、管理和运营体制机制，制定产业扶持政策，做好特区部分重大项目申报筹备，启动重点片区和重大项目招商推介工作，尽快拉开曲阜文化经济特区建设框架。做好文化遗产保护工作，加快推进孔孟文化遗产地、孔子博物馆、“三孔”古建筑油饰彩绘保护、鲁国故城考古遗址公园等重点项目。搞好孔子学院总部体验基地规划建设。以全球视野和国际标准，策划一批体验项目，全方位提升服务设施功能，加强与世界各地孔子学院、孔子课堂的互动交流，力争年内完成体验基地建设前期任务。实施孔孟之乡优秀传统文化研究和传播人才专项工程，打造儒学研究高地，提升儒学研究交流话语权。做大做强文化产业。深化文艺院团和旅游管理体制改革，充分运用市场机制配置各类要素资源，推动文化产业与科技、金融深度融合。加大文化产业招商力度，吸引一批文化产业战略投资者和企业参与我市文化建设。加快曲阜国家级文化产业示范园、嘉祥石雕文化产业园、梁山出版印刷产业园等特色文化园区转型升级。积极培育文化创意、演艺娱乐、数字动漫等新兴文化产业。构建公共文化服务体系。加快市文化中心“一体四馆”建设，完成杂技城主体工程和配套设施。开展基层公共文化服务中心建设试点，农村文化广场实现行政村全覆盖。常态化实施“千场大戏进农村”、“万场演出惠民生”、农村公益电影放映等文化惠民工程，丰富人民群众精神文化生活。

（九）大力发展社会事业，全方位提升百姓福祉。心怀群众冷暖疾苦，多思群众所想所盼，多谋利民惠民之事，继续办好为民10件实事，切实做好保障和改善民生各项工作。

推动更高质量的充分就业。坚持以创业带就业，实施万人创业助推工程，设立小额担保贷款公共服务中心，落实创业培训补贴、小额担保贷款及贴息、税费减免等政策，降低自主创业门槛，扶持更多劳动者就业创业。搞好就业培训，保障劳动者权益，开发更多公益性岗位。年内开展职业技能培训6万人以上，扶持创业1万人以上，新增城镇就业6万人，农村劳动力转移就业7.1万人，高校毕业生就业率达到85%以上。

办好人民更加满意的教育。顺应群众追求优质教育需求，大力发展学前教育，加快推进县域义务教育均衡发展，持续提升高中教育水平。全面改善义务教育薄弱学校基本办学条件，新改扩建城乡幼儿园60所、城区校舍17万平方米、农村校舍40万平方米。继续实施市区教育重点项目建设，新增办学规模110个班。扩大“学生营养改善计划”覆盖面，将全市农村中小学生全部纳入补助范围。实施“名师、名校长”工程，多种形式推进校长、教师城乡双向交流。扎实开展“康教结合”实验工作，提高特殊教育水平。发展现代职业教育，支持驻济高校围绕产业需求设置专业、培养人才，促进教育链与产业链有机融合，增强教育服务经济社会发展能力。

构筑更加公平完善的社保体系。强化社会保险扩面征缴，实施“全民参保登记计划”，推动社保工作由制度全覆盖向人员全覆盖转变。做好企业基本养老保险待遇调整工作，推进机关事业单位养老保险制度改革，解决好被征地农民和采煤塌陷失地农民的养老保障问题。持续提升社会保险待遇水平，城乡居民基础养老金标准提高至每人每月85元，城乡居民和城镇职工基本医疗保险、大额医疗救助最高报销额均提高到35万元。进一步提高城乡最低生活保障标准，主要救助指标高于全省平均水平。实施系列惠残助残行动，继续抓好白内障无障碍和残疾儿童免费抢救性康复等重点项目，加快残疾人康复培训基础设施建设，打造“幸福港湾”。

提供更加优质的医疗卫生服务。实施妇幼健康科技惠民工程，建立在校中小学生健康查体制度。改进和完善计划生育管理体制，开展出生人口性别比综合治理。加强和规范乡镇卫生院管理，落实乡村医生待遇。实施新一轮中医药特色能力提升工程，满足多样化群众就医需求。年内市第一人民医院高新区分院建成使用，市中医院新院区主体工程竣工。

建立更加科学的住房保障体系。健全以公共租赁住房为主体的住房保障制度，新建公租房3000套，改造棚户区4.95万户，完成省下达的农村危房改造任务。扩大住房公积金制度覆盖面。完善保障性住房建设、审核、分配办法，探索推行租补分离，落实配套监管措施，强化动态跟踪管理，健全退出机制，确保保障性住房分配管理公平公正。

构建更加有效的社会治理体系。牢固树立依法治理理念，大力推行社会治理网格化模式，将矛盾纠纷调处、治安隐患排查、社情民意研判、风险稳定评估、流动人员管理全部纳入网格管理。探索建立社区、社工、社会组织和志愿者联动机制，完善以群众自治组织为主体，社会广泛参与的新型城乡社区治理体系。深入推进平安济宁建设，健全立体化社会治安防控体系，严密防范、严厉打击各类违法犯罪活动。落实领导干部接访包案制度，促进矛盾及时有效化解。强化安全生产责任落实，深入排查治理安全隐患，加大安全执法监管，严厉打击非法违法生产经营行为，保持安全生产形势持续稳定。加强食品药品安全治理能力建设，强化“餐桌污染”监管，打造食品药品消费安全放心城市。加强人民调解、法律援助和社区矫正工作，创建省级劳动关系和谐城市。圆满完成争创全国双拥模范城“七连冠”目标。加强国防动员、人民防空、民兵预备役建设。统筹做好民族宗教、外事侨务、对台和老龄工作，提升物价、统计、体育、水文、防汛、气象、史志和防震减灾等项工作水平。

三、全面加强政府自身建设

巩固扩大党的群众路线教育实践活动成果，不断改进工作作风，以敢于担当的精神、攻坚克难的勇气，切实肩负起新常态下抓住新机遇、实现新发展的重任。

进一步提高依法行政能力。按照“法无授权不可为”的原则，严格按照法定权限和程序行使权力、履行职责、管理事务，努力建设执法严明、廉洁高效的法治政府。制定政府权力清单、责任清单和市场准入负面清单，消除权力设租寻租空间，坚决纠正不作为、乱作为，坚决克服懒政、怠政，坚决惩处失职、渎职。坚持重大行政决策公众参与、专家论证、风险评估、合法性审查、集体讨论决定法定程序，积极推行政府法律顾问制度，建立重大决策终身责任追究及责任倒查机制，提高政府行政决策的科学化、民主化水平。督促引导各级政府及其工作人员，带头尊法学法守法用法，提高运用法治思维和法治方式履行职责的能力和水平。自觉接受人大法律监督、工作监督和政协民主监督，扎实做好人大代表建议、政协提案办理工作，广泛接受社会各方面的监督。

进一步提高政务服务能力。创新政府管理服务方式，优化部门职能配置，所有承担行政审批职能的部门全部实行“一个窗口”对外统一受理，所有审批事项全部编制服务指南，明确申请条件、基本流程、示范文本，绝不让企业和群众摸不清门、跑累了腿；推行“限时办理”，健全受理单制度和办理时限承诺制，探索建立“一条龙”审批或并联审批；严格“规范办理”，避免随意裁量行为；坚持“透明办理”，强化办理过程信息公开、内部督查和社会监督，杜绝暗箱操作；积极推进“网上办理”，实行网上预受理、预审查，努力打造行政审批“项目最少、程序最简、效率最高”的政府服务品牌。加大政务公开力度，推进决策公开、执行公开、管理公开、服务公开、结果公开，确保权力在阳光下运行。完善政府信息发布制度，充分利用新闻发布会、政府门户网站、政务微博微信和“12345”市长公开电话等平台，及时回应群众利益关切、答复公众诉求，搭建起政府联系群众的桥梁和纽带。

进一步提高真抓实干能力。改进会风文风，精简会议文件，把更多精力放到研究工作、谋划发展上来。坚持问题导向，紧扣发展主题，对制约发展的瓶颈，一个一个地研究，一个一个地突破。坚持以身作则、率先垂范，深入建设一线、项目现场，努力把问题解决在一线、任务完成在一线。深化拓展领导驻村、干部联系农户活动，多到困难矛盾多、群众意见大的地方去，以抓铁有痕的毅力、亲民为民的情怀，着力解决关系群众切身利益的实际问题。健全行政绩效管理，严格督查考核，严明工作纪律，坚决治理“为官不为”，不断增强政府公信力和执行力。

进一步提高拒腐防变能力。坚决执行中央“八项规定”和省、市委实施办法，持之以恒反对“四风”；严格执行党政机关厉行节约反对浪费条例和公务接待规定，加强“三公经费”监管，严禁超预算支出，努力建设节约型机关。严格落实党风廉政建设责任制，抓好主体责任落实，做到守土有责、守土尽责。扎实推进惩治和预防腐败体系建设，完善防止权力寻租和利益输送的体制机制。强化监察、审计监督和政府内部层级监督，严肃追究失职渎职行为，严厉查处各种违法违纪案件，坚决纠正损害群众利益的不正之风，以为民、务实、清廉的良好形象赢得人民群众的信任和支持。

各位代表！新的一年蕴含新的机遇，新的征程充满新的希望。让我们更加紧密地团结在以习近平同志为总书记的党中央周围，在省委、省政府和市委的坚强领导下，紧紧依靠全市人民，坚定信心，振奋精神，锐意进取，扎实工作，为打造鲁西科学发展高地、确保与全省同步提前全面达小康而努力奋斗！
